# The Functional Significance of the Rho/Rho-Kinase Pathway in Human Erythrocytes

**DOI:** 10.4274/tjh.2013.0115

**Published:** 2014-06-10

**Authors:** R. Nalan Tiftik, Oğuz K. Başkurt, Seval Kul, Kansu Büyükafşar

**Affiliations:** 1 Mersin University Faculty of Medicine, Department of Pharmacology, Mersin, Turkey; 2 Koç University Faculty of Medicine, İstanbul, Turkey; 3 Gaziantep University Faculty of Medicine, Department of Biostatistics, Gaziantep, Turkey

**Keywords:** Erythrocyte deformability, RhoA, Rho-kinase, Y-27632, Fasudil, Lysophosphatidic acid, C3

## Abstract

**Objective:** Erythrocyte deformability, which can be influenced by various intracellular signaling mechanisms, such as nitric oxide, cAMP, cGMP, and protein kinases, is the most important physiological factor providing the blood flow in microcirculation. However, the functional significance of the Rho/Rho-kinase pathway, which contributes cell shape changes and the reorganization of the actin cytoskeleton, has yet to be explored in erythrocytes. Therefore, we examined the influence of several activators and inhibitors of Rho/Rho-kinase signaling on human erythrocyte deformability.

**Materials and Methods:** RhoA and ROCK-2 proteins were studied by western blotting. Influences of 2 Rho-kinase inhibitors, fasudil and Y-27632 (both 10-7 to 10-4 M), on erythrocyte deformability was determined by ektacytometer at various shear stresses (0-30 Pa) in the presence or absence of a known Rho activator, lysophosphatidic acid (LPA, 10-5 to 5x10-5 M, 1-15 min).

**Results: **LPA incubation reduced deformability with concomitant RhoA-GTP inhibition. Y-27632 and fasudil also decreased deformability, but had no effect on LPA-induced reduction of deformability. Rho inhibitor C3 had no effect on RhoA activation. Reduction in RhoA activation was induced by sub-hemolytic mechanical stress.

**Conclusion:** Our findings may indicate that the Rho/Rho-kinase pathway could contribute to the regulation of deformability of human erythrocytes.

## Abstract (tr)

**Amaç:** Nitrik oksid, sAMP, sGMP ve protein kinazların rol oynadığı çok çeşitli hücre içi sinyal mekanizmasının etkileyebildiği eritrosit deformabilitesi mikrosirkülasyonun devamlılığında rol oynayan en önemli fizyolojik faktördür. Ancak, hücre şekil değişiklikleri ve aktin hücre iskeleti reorganizasyonu gibi hücresel olaylara aracılık eden Rho/Rho-kinaz yolağının eritrositlerdeki fonksiyonel önemi henüz tam olarak ortaya konulmamıştır. Bu yüzden, söz konusu sinyal ileti mekanizmasının çeşitli aktivatör ve inhibitörlerinin eritrosit deformabilitesi üzerine etkisini inceledik.

**Gereç ve Yöntemler: **Eritrositlerde RhoA ve ROCK-2 proteinleri Western-Blot yöntemi ile gösterildi. Her iki Rho-kinaz inhibitörü fasudil ve Y-27632’nin (10-7-10-4 M) eritrosit deformabilitesi üzerine etkisi çeşitli sıvı kayma kuvvetlerinde (0-30 Pa) bilinen bir Rho aktivatörü olan lizofosfatidik asit (LPA, 10-5- 5x10-5 M, 1-15 dakika) varlığında ve yokluğunda incelendi.

**Bulgular:** LPA inkübasyonu Rho-GTP inhibisyonu ile eşzamanlı olarak deformabiliteyi azalttı. Y-27632 ve fasudil de tek başlarına deformabiliteyi azalttı ancak LPA ile indüklenen deformabilite azalmasını etkilemedi. Rho inhibitörü C3 ekzoenzim RhoA aktivasyonunu etkilemedi. Öte yandan subhemolitik mekanik stress uygulaması RhoA aktivasyonunu azalttı.

**Sonuç:** Sonuç olarak bulgularımız Rho/Rho-kinaz yolağının insan eritrositlerinde deformabilitenin düzenlenmesine katkı yapabileceğine işaret edebilir.

## INTRODUCTION

The maintenance of normal deformability and mechanical stability is critical for human erythrocytes, which undergo extensive deformations in the microvasculature, to perform their function of oxygen delivery during their lifespan [1]. Enzymes associated with the erythrocyte membrane are known to have important roles in regulating erythrocyte shape and deformability [2]. Moreover, the L-arginine-NO pathway; membrane proteins such as actin, α- and β-spectrin, adducin, and dematin; ion pumps like Na+-K+ ATPase and Ca2+-Mg2+ ATPase; and second messengers like cAMP and cGMP provide direct/indirect contributions to the regulation of erythrocyte deformability [3,4,5,6]. 

The Rho/Rho-kinase (ROCK) pathway, one of the most widely studied cell signaling pathways recently, takes part in smooth muscle cell contraction via a phenomenon called Ca+2 sensitization [7,8,9,10,11,12,13] and mediates fundamental cellular functions in non-muscle cells [14]. These functions include stress fiber formation, membrane ruffling, cytokinesis and cell migration, actin cytoskeleton reorganization, proliferation, hypertrophy, cell shape changes, platelet aggregation, and lymphocyte and fibroblast adhesion [14,15,16]. Furthermore, the Rho/ROCK pathway is involved in some physiological and/or pathological processes such as vasoconstriction, hypertension, coronary artery spasm, and ischemia-reperfusion injury of the heart [17,18,19,20]. With regard to blood cells, Rho signaling mediates several cellular events in platelets, neutrophils, and lymphocytes, such as chemotaxis, cell shape changes, and the secretion functions [21,22,23,24].

The Rho protein has been detected in both cytosol and membrane fractions of erythrocytes and was found to bind to the cytoplasmic surface of the cell membrane with high affinity [25]. In addition, PRK1/PKN, a cytosolic serine/threonine kinase, which was previously described as one of the RhoA effectors, is localized in the erythrocyte plasma membrane [26].

However, the possible contribution of Rho/ROCK signaling to the physiological control of erythrocyte deformability has yet to be investigated in human erythrocytes. 

Therefore, in this study we aimed to examine the influence of 2 Rho-kinase inhibitors, fasudil and Y-27632, and Rho activator lysophosphatidic acid (LPA) on erythrocyte mechanical properties. This was done using an ektacytometer, which is currently the most widely used and most reliable approach to the measurement of erythrocyte deformability [27].

## MATERIALS AND METHODS

**This study was approved by the Mersin University Clinical Research Ethics Committee.**

**Blood Sampling **

Blood was drawn by venipuncture into heparinized (15 IU/mL) syringes from healthy adult male volunteers aged between 18 and 45 years. Erythrocytes were isolated from whole blood by centrifugation (3000 rpm, 10 min) followed by 3 washing steps in phosphate-buffered saline (PBS) to remove leukocytes. Thereafter, the washed erythrocytes were resuspended in autologous plasma at a hematocrit level of 40%. The samples were studied within 4-6 h after the venipuncture. Erythrocytes used for western blotting were prepared by the same procedure. 

**Chemicals**

(+)-(R)-trans-4-(1-Aminoethyl)-N-(4-pyridyl) cyclohexanecarboxamide dihydrochloride monohydrate (Y-27632), fasudil (HA-1077), and oleoyl-L-lysophosphatidic acid sodium salt (LPA) were obtained from Sigma (St. Louis, MO, USA). Y-27632 and fasudil were dissolved in saline. LPA was diluted in PBS. 

**Western Blot Analysis**

Isolated erythrocytes were lysed in equal volumes of hypotonic lysis buffer solution (composed of 10 mM Tris, pH 7.6; 1 mM EDTA; and 20 µg/mL phenylmethylsulfonyl fluoride) as described previously [28]. Membranes were recovered by centrifugation at 38.000 x g for 90 min. The supernatant was preserved as the cytosolic fraction. Both the pellet containing the membranous fraction and the supernatant were separately collected and preserved at -20 °C until being used for protein analysis by the Bradford method and western blot analysis. Equal amounts of protein were loaded into wells, separated by electrophoresis on 10% polyacrylamide-sodium dodecyl sulfate gel, and then transferred to a nitrocellulose membrane. Thereafter, the membranes were blocked with fatless milk powder in Tris-buffered solution containing 0.05% Tween-20 for 1 h. They were then probed with primary antibodies raised against RhoA (mouse monoclonal, Santa Cruz Biotechnology Inc., Santa Cruz, CA, USA) at 1:500 dilution and ROCK-2 (polyclonal IgG, Santa Cruz Biotechnology Inc.) at 1:2000 dilution, followed by horse radish peroxidase-conjugated secondary antibody (goat anti-mouse and donkey anti-goat, 1:2000, Santa Cruz Biotechnology Inc.). The blots were then assayed with an enhanced chemiluminescence detection kit (ECL Plus, Amersham Biosciences, Freiburg, Germany) and visualized on a commercial X-ray film.

**Effects of Rho/ROCK Activators and Inhibitors on Erythrocyte Deformability**

LPA (10-5 M, 2x10-5 M, 5x10-5 M) was added to the erythrocyte suspensions and incubated for various periods of time (1, 2, 5, 10, and 15 min) at room temperature, and erythrocyte deformability was measured afterwards. In another series of experiments, the erythrocyte suspensions were preincubated with 10-5 to 10-4 M Y-27632 or fasudil for 45 min before incubation with LPA (10-5 M for 10 min). In another group, erythrocytes were treated with exoenzyme C3 transferase from Clostridium botulinum (3 µg/mL for 4 h; CT04-B, Cytoskeleton, Denver, CO, USA). All control suspensions were incubated with their own vehicles.

Erythrocyte deformability was determined at various fluid shear stresses by laser diffraction analysis using an ektacytometer (LORCA, RR Mechatronics, Hoorn, the Netherlands). Following the incubation processes as described above, erythrocyte suspensions were diluted in 6% polyvinylpyrrolidone (MW: 360.000; Sigma, St. Louis, MO, USA) solution prepared in PBS at a ratio of 1:200. The details of the measurement system have been described elsewhere [29].

One milliliter of this suspension was sheared in a Couette system composed of a glass cup and a precisely fitting bob, with a gap of 0.3 mm between the cylinders. A laser beam was directed through the sheared sample, and the diffraction pattern produced by the deformed cells was analyzed by a microcomputer. On the basis of the geometry of the elliptical diffraction pattern, an elongation index (EI) was calculated: EI= (L–W) / (L+W), where L and W are the length and width of the diffraction pattern, respectively. EI values were determined for 9 shear stresses between 0.3 and 30 Pa. All measurements were carried out at 37 °C. Additionally, the shear stress required for half-maximal deformation (SS1/2) and maximum elongation index (EImax) were calculated from the data set for each measurement by using a Lineweaver-Burk analysis procedure [29].

**ROCK Inhibitors and Erythrocyte Mechanical Damage **

Erythrocytes pretreated with Y-27632 (10-5 to 10-4 M) or fasudil (10-5 to 10-4 M) for 1 h in autologous plasma were exposed to sub-hemolytic mechanical stress of 120 Pa for 30 s. Erythrocyte suspensions were diluted in 25% dextran 40 (40.6 kDa, Sigma Chemical Co.) solution, with 19.5 mPas viscosity at 37 °C, measured by a cone-plate viscometer (Wells-Brookfield, Brookfield Engineering Labs, Middleboro, MA, USA). Erythrocyte suspensions were loaded into the Couette shearing system described above (LORCA) and the outer cylinder was rotated at a rotational speed calculated to obtain 120 Pa of shear stress. The details of the mechanical shearing procedure were described elsewhere [30]. Erythrocyte suspensions in the viscous medium were exposed to shear stress for 30 s at 37 °C. Following the shearing period, the erythrocyte suspensions were directly used for erythrocyte deformability testing as described above. Control samples were not exposed to the 120 Pa of shear stress; instead, they were suspended in the 25% dextran 40 solution and kept in the solution for as long as the corresponding sheared samples before being used in the deformability measurements.

**RhoA Activation Assay**

The active Rho GTP levels of erythrocytes incubated with LPA (10-5 M for 1, 2, 5, 10, and 15 min), exposed to mechanical stress (120 Pa, 30 s) and treated with exoenzyme C3 transferase from Clostridium botulinum (3 µg/mL for 4 h), were evaluated by colorimetric G-LISA activation assay kit (BK124, Cytoskeleton). 

**Statistics**

All data are represented as mean±standard error (SE) of the mean. Statistical comparisons were done by paired t-test or Wilcoxon signed-rank test after the Kolmogorov-Smirnov test was used for normality of results. In addition, a one-sample t-test was used for % RhoA activation in LPA series. P-values of less than 0.05 were considered significant.

## RESULTS

**The Expression of RhoA and ROCK Proteins in Erythrocytes **

**Figure 1:**
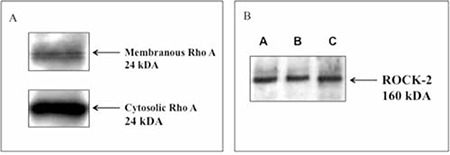
Western blotting for RhoA (A) and Rho-kinase (ROCK-2) (B) in human erythrocytes. Both cytosolic and membranous fractions for RhoA (MW: 24 kDa) and cytosolic fraction for ROCK-2 (MW: 160 kDa) were studied. A, B, and C are erythrocytes isolated from different volunteers.

Western blotting analysis revealed that both RhoA and ROCK-2 proteins were expressed in human erythrocytes. There were RhoA expressions in both membranous and cytosolic fractions (Figures 1A and 1B).

**Effects of LPA and ROCK Inhibitors, Y-27632 and Fasudil, on Erythrocyte Deformability**

**Figure 2:**
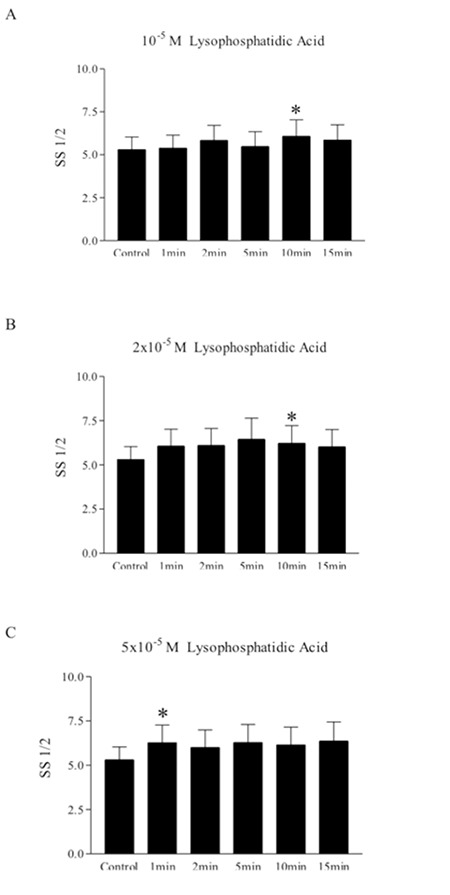
Effect of lysophosphatidic acid (10-5, 2x10-5, 5x10-5 M) on shear stress for half-maximal deformation (SS1/2) at different time periods. Data are mean ± SE, n=8. Statistical analyses were done by Wilcoxon signed ranks test (*: p<0.05, difference from control).

**Figure 3:**
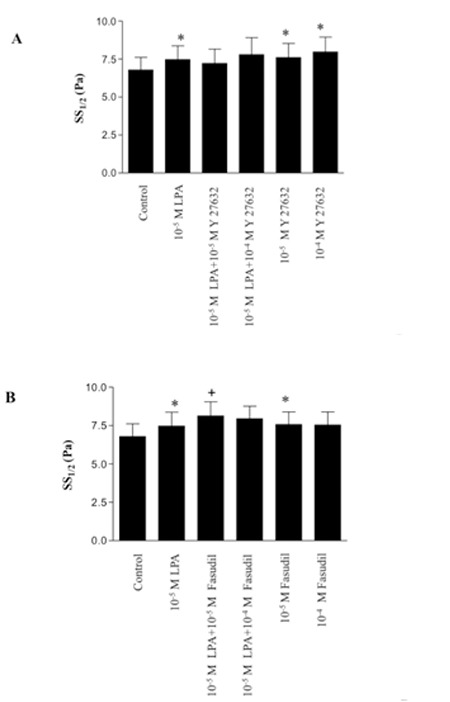
The potential effect of Y-27632 (A) and fasudil (B) on LPA-induced deformability. Data are mean ± SE, n=9. Statistical analysis was done by Wilcoxon signed ranks test (*: p<0.05, difference from control; +: p<0.05, difference from LPA).

LPA (10-5 to 5x10-5 M, incubated for 1-15 min) decreased erythrocyte deformability, as indicated by increased SS1/2 values evaluated at different time points (Figures 2A-2C). Similarly, ROCK inhibitors Y-27632 (10-5 to 10-4 M) and fasudil (10-5) also increased SS1/2 values, thus demonstrating decreased deformability. However, neither Y-27632 (10-5 to 10-4 M, Figure 3A) nor fasudil (10-4 M, Figure 3B) in combination with LPA had additional effects on deformability reduction. 

**Effects of Y-27632 and Fasudil on Mechanical Stress-Induced Deformability Changes**

Mechanical stress (120 Pa, 30 s) deteriorated erythrocyte deformability, but neither Y-27632 nor fasudil (10-4 to 10-5 M) had effects on the impaired deformability (data not shown).

**Rho-GTP Levels of the Erythrocytes Exposed to Mechanical Stress, LPA, or Clostridium botulinum Exoenzyme C3 Transferase**

**Figure 4:**
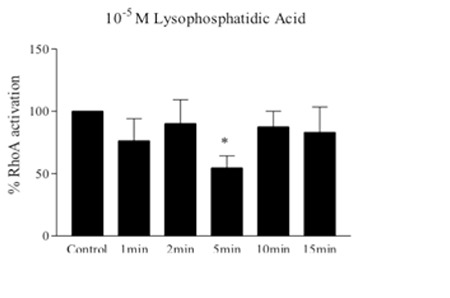
Effect of lysophosphatidic acid (10-5 M) on RhoA activation of erythrocytes. Rho-GTP levels were measured with G-LISA activation assay kit and expressed as % RhoA activation. Data are mean ± SE, n=8. Statistical analysis was done by one-sample t-test (*: p<0.05, difference from control).

**Figure 5:**
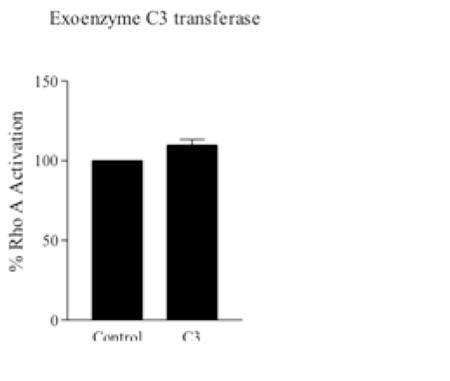
Rho-GTP level in erythrocytes treated with exoenzyme C3 transferase (3 µg/mL). Rho-GTP levels were measured with G-LISA activation assay kit and expressed as % RhoA activation. Data are mean ± SE, n=5. Statistical analysis was done by one-sample t-test (p=0.0526).

**Figure 6:**
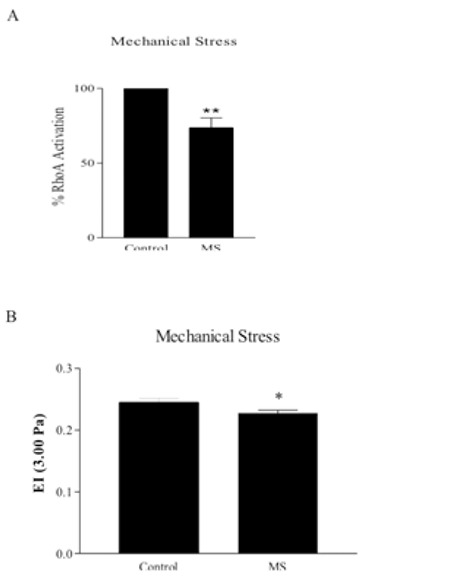
A) Rho-GTP level in erythrocytes exposed to mechanical stress (120 Pa, 30 s). Rho-GTP levels were measured with G-LISA activation assay kit and expressed as % RhoA activation. Data are mean ± SE, n=4. Statistical analyses were done by one-sample t-test (**: p<0.01, difference from control). B) Mechanical stress induced deterioration of deformability. Data are mean ± SE, n=8. Statistical analysis was done by paired t-test (*: p<0.05, difference from control).

A well-known Rho activator, LPA (10-5) surprisingly reduced the Rho-GTP levels in erythrocytes at 5 min of incubation (Figure 4). To ensure this inhibitor effect of LPA on RhoA activation, we tentatively evaluated the activation of RhoA in exoenzyme C3 transferase-treated red blood cells by colorimetric G-LISA activation assay kit, and C3 tended to increase RhoA activation (Figure 5). In the erythrocytes under mechanical stress (120 Pa, 30 s), Rho-GTP levels significantly decreased (Figure 6A) and deformability was also impaired (Figure 6B).

## DISCUSSION

RhoA protein was demonstrated to be expressed both in cytosolic and membranous fractions of erythrocytes, and cytosolic fraction is known to translocate to the plasma membrane [25,31]. Moreover, it has also been reported that ROCK-1 enzyme-dependent myosin-mediated contractions are necessary for caspase activation of phorbol ester-induced apoptosis in an erythrocyte precursor, the TF-1 cell line [32]. We also demonstrated RhoA protein both in the cytosol and membrane of erythrocytes, confirming the results of the above studies. In addition to this, we further demonstrated ROCK-2 protein expression in human erythrocytes by western blotting. 

A membrane phospholipid, LPA is known to activate Rho proteins [33] through specific receptors coupled with heterotrimeric G proteins that mediate several biological signals. LPA has 5 different receptors identified so far (LPA1-LPA5) and is known to have a role in cell shape changes and contractility via LPA1 and LPA2 receptors by activating the downstream Rho through the activation of heterotrimeric G protein Gα12/13 in cells such as smooth muscle cells and neuronal cells [33,34]. LPA is a widespread tool that is used for exploring the consequences of RhoA activation in most studies to understand the possible contribution of the Rho/Rho-kinase pathway to cell functions in non-muscle cells. Surprisingly enough, LPA inhibited RhoA activation in erythrocytes in our study. The reason why it reduced RhoA translocation might be because its receptors present on human erythrocytes could be coupled with RhoA inhibition, in contrast to those in other cells. LPA may also activate protein kinase C [35], which can increase intracellular Ca2+ concentration [Ca2+i]. If the fact that LPA attenuates RhoA activation and concomitantly reduces deformability is the case, this may be physiologically important as LPA is naturally present in cell membranes [36]. Maintenance of the [Ca2+i] of erythrocytes is of essential importance since any tiny oscillation in [Ca2+i] may result in dramatic deformability changes [37]. Thus, calcium sensitizing pathways such as Rho/ROCK and protein kinase C seem to be especially important in erythrocyte physiology. 

In addition to the detection of Rho expression in human erythrocytes, we also demonstrated ROCK expression and tested the effects of inhibitors of this protein on erythrocyte mechanical properties, namely Y-27632 and fasudil. Y-27632 and fasudil did not produce an additional effect on LPA-induced impairment of deformability. However, the ROCK inhibitors decreased deformability on their own. This finding could indicate the functional importance of this pathway in erythrocyte deformability. However, the recent report of Thuet et al. (2011) had opposite findings, such that ROCK inhibition may improve erythrocyte deformability. This contradiction might be due to the usage of different methods; Thuet et al. measured erythrocyte deformability by using a blood filtrometer. Currently, the ektacytometer is the more widely used and more reliable technique for the measurement of erythrocyte deformability as compared to blood filtration methods [27]. Erythrocyte filterability can be seriously affected by various cellular properties of erythrocytes apart from deformability, e.g., mean corpuscular volume [38] and the presence of leukocytes and platelet aggregates in samples [39]. Ektacytometry is not influenced by such artifacts and is much more sensitive to changes in erythrocyte deformability [27].

On the other hand, sub-hemolytic mechanical trauma leads to changes in cellular metabolism, membrane organization, ion transport, and cell membrane rheological properties in erythrocytes [40]. Therefore, in our study, we also tested the possible role of the Rho/ROCK pathway in the deformability impairment induced by mechanical stress (120 Pa for 30 s). This mechanical stress application was previously shown to cause a deterioration of deformability of the erythrocytes [30]. Interestingly, in the present study the mechanical stress not only impaired the deformability but also diminished RhoA protein activation. 

We also evaluated RhoA activation after C3 incubation and, surprisingly, RhoA activation tended to increase. This was absolutely unexpected since C3 is widely known as a Rho inhibitor. These observations need to be confirmed by further studies, to clarify if RhoA activation rather than inhibition is specific to erythrocytes. 

In conclusion, we had 4 consistent findings in favor of Rho/ROCK signaling as a factor affecting erythrocyte deformability: 1) LPA decreased RhoA activation while it impaired erythrocyte deformability; 2) erythrocyte deformability was also impaired under the influence of ROCK inhibitors, whereby Y-27632 and fasudil decreased deformability; 3) botulinum toxin C3 tended to increase the activation of RhoA; 4) mechanical stress, which decreased deformability, also caused Rho inhibition. The Rho/ROCK signaling cascade could play an important role in the regulation of human erythrocyte deformability. These findings contribute to the understanding of the intracellular signaling pathways of active regulation of erythrocyte deformability, with potential implications for the erythrocyte-centered regulation of microcirculatory blood flow. 

## ACKNOWLEDGMENTS

This study was carried out as part of a PhD thesis by R. Nalan Tiftik, presented to the Mersin University Health Sciences Institute. This study was supported by Mersin University (BAP-SBE-FB (RNT) 2007-2DR). The authors are indebted to Murat Uyuklu and Pınar Ülker from the Department of Physiology, Faculty of Medicine, Akdeniz University, and Özge Güldalı-Dutlu from the Department of Medical Pharmacology, Faculty of Medicine, Mersin University, for their help. 

## CONFLICT OF INTEREST STATEMENT

The authors of this paper have no conflicts of interest, including specific financial interests, relationships, and/ or affiliations relevant to the subject matter or materials included.
